# Insights into the unique roles of dermal white adipose tissue (dWAT) in wound healing

**DOI:** 10.3389/fphys.2024.1346612

**Published:** 2024-02-23

**Authors:** Yu Li, Jie Long, Ziang Zhang, Wen Yin

**Affiliations:** Xijing Hospital, Air Force Medical University, Xi’an, Shaanxi, China

**Keywords:** wound healing, dermal white adipose tissue, fibrosis, ECM, immune response, scar

## Abstract

Dermal white adipose tissue (dWAT) is a newly recognized layer of adipocytes within the reticular dermis of the skin. In many mammals, this layer is clearly separated by panniculus carnosus from subcutaneous adipose tissue (sWAT). While, they concentrated around the hair shaft and follicle, sebaceous gland, and arrector pili muscle, and forms a very specific cone geometry in human. Both the anatomy and the histology indicate that dWAT has distinct development and functions. Different from sWAT, the developmental origin of dWAT shares a common precursor with dermal fibroblasts during embryogenesis. Therefore, when skin injury happens and mature adipocytes in dWAT are exposed, they may undergo lipolysis and dedifferentiate into fibroblasts to participate in wound healing as embryogenetic stage. Studies using genetic strategies to selectively ablate dermal adipocytes observed delayed revascularization and re-epithelialization in wound healing. This review specifically summarizes the hypotheses of the functions of dWAT in wound healing. First, lipolysis of dermal adipocytes could contribute to wound healing by regulating inflammatory macrophage infiltration. Second, loss of dermal adipocytes occurs at the wound edge, and adipocyte-derived cells then become ECM-producing wound bed myofibroblasts during the proliferative phase of repair. Third, mature dermal adipocytes are rich resources for adipokines and cytokines and could release them in response to injury. In addition, the dedifferentiated dermal adipocytes are more sensitive to redifferentiation protocol and could undergo expansion in infected wound. We then briefly introduce the roles of dWAT in protecting the skin from environmental challenges: production of an antimicrobial peptide against infection. In the future, we believe there may be great potential for research in these areas: (1) taking advantage of the plasticity of dermal adipocytes and manipulating them in wound healing; (2) investigating the precise mechanism of dWAT expansion in infected wound healing.

## 1 Introduction

Adipose tissue is one of the largest endocrine organs in the human body, playing a crucial physiological role. It regulates metabolism and immune system function throughout the body. Although most adipose tissue is located subcutaneously and functions similarly, each adipose tissue depot has unique functions and characteristics based on its distinct distribution. Currently, adipose tissue is primarily classified into three main types based on its distribution and function: White Adipose Tissue (WAT): Widely distributed throughout the body, it serves as the primary site for energy storage, with an insulation function to regulate body temperature. Brown Adipose Tissue (BAT): Mainly found around the neck, shoulder blades, and areas of the upper back, brown adipose tissue contains more mitochondria and brown adipocytes capable of generating heat. It is a major source of thermogenesis, contributing to the maintenance of body temperature. Beige Fat is a special type of adipose tissue discovered through biomedical research and imaging techniques. It is mainly located around the neck, chest, and lumbar region, exhibiting characteristics of both brown and white adipose tissue. Beige fat contains mitochondria that contribute to heat production and promote fat metabolism and breakdown.

Beyond the well-defined adipose distributions mentioned above, Querleux B (Querleux et al., 2002) utilized high spatial resolution magnetic resonance imaging to characterize the three-dimensional structure of the tissue at the junction between the dermis and subcutaneous fat, as well as the subcutaneous fibrous septa. A distinct type of fat, different from that in subcutaneous tissue, was identified in the dermal adipose tissue, establishing it as a unique type of white adipose tissue. Subsequently, Zhuzhen Z and their team (Zhang et al., 2019a) successfully isolated and cultured a unique type of white adipose cells within the dermal tissue of mouse skin, characterizing dermal adipose at the cellular and molecular levels. Through a combination of pulse-chase lineage tracing and single-cell RNA sequencing, mature dermal adipose cells were observed to undergo dedifferentiation and redifferentiation under physiological and pathological conditions, defining them as a unique type of adipose cell capable of reversible dedifferentiation and redifferentiation. Multiple studies have observed the presence of this unique fat distribution located beneath the reticular dermis, subsequently defined as dermal white adipose tissue (dWAT). Interestingly, it was found that, functionally, dWAT exhibits superior responsiveness to various external stimuli compared to subcutaneous white adipose tissue. For example, during the hair follicle cycle, the size and number of dermal adipose cells increase with follicle growth, while they significantly decrease with follicle regression (Kruglikov and Scherer, 2016). Zhang LJ (Zhang LJ. et al., 2015) demonstrated that following *Staphylococcus aureus* infection, there is a rapid proliferation of preadipocytes in the dermis and expansion of the dermal fat layer. Conversely, impaired fat generation leads to an increased incidence of skin infections. Kasza (Kasza et al., 2016) conducted imaging studies using an MRI scanner on control and high-fat diet-fed mice to investigate fat accumulation in specific fat cell depots. Measurements of adipose tissue volume showed an initial expansion of dermal adipose volume within the first 3 weeks, and in the group of mice on a high-fat diet, dWAT thickness was greater than that in the control group. Kasza I, Suh Y, and others (Kasza et al., 2014) demonstrated that mice with intact dWAT could appropriately respond to cold stress. Experimental studies revealed that when mice were placed in a ‘room temperature’ environment (21°C–24°C/70–75°F), dWAT thickened accordingly, while transitioning mice to a warm environment (29°C–33°C/84–91°F) resulted in a thinning of dWAT volume. These findings suggest that this particular fat depot exhibits a highly dynamic response to different external factors.

Dermal White Adipose Tissue (dWAT), in addition to responding to various external stimuli with changes in volume, undergoes significant alterations during the body’s repair processes following injury, ultimately influencing wound healing outcomes. Barbara A (Schmidt and Horsley, 2013a). Schmidt utilized immunostaining with antibodies against the adipocyte marker perilipin A to demonstrate the presence of small perilipin A+ adipocytes in wounds 5 days after re-epithelialization. This indicates that during the wound healing stage, dWAT reappears in skin wounds, actively participating in wound repair. Furthermore, Stepp, M. A (Stepp et al., 2002) found that in Sdc1 −/− mice lacking dWAT, there were effects on epithelial cell proliferation and regulation of alpha9 integrin expression, impacting the generation of mature dWAT and influencing tissue regeneration during the wound repair phase, ultimately resulting in impaired wound healing. In addition, Driskell, R. R (Driskell RR. et al., 2013) demonstrated that inhibiting dWAT generation during the repair phase of wound healing reduces the ability of fibroblasts to refill the wound bed and severely affects the wound healing process. These experiments illustrate that dWAT, through its dynamic changes and regulation of the functions of surrounding cells, actively participates in the wound healing process. The absence or impaired function of dWAT directly leads to poor wound healing. However, the specific mechanisms and potential functions through which dWAT affects the wound healing process remain unclear.

As a relatively novel yet highly intriguing area of study, dWAT holds extensive research prospects and clinical application potential. This review focuses on the distribution, differentiation, and formation of dWAT; tissue specificity; and delves into the immune response, skin fibrosis, and scar formation aspects of dWAT in the wound healing process. Exploring the functions and mechanisms of dWAT not only enhances our understanding of the pathology and disease mechanisms of dWAT in wound healing but also provides new perspectives and opportunities for future skin disease treatments and wound healing strategies.

## 2 The anatomy of dWAT

### 2.1 Distribution of dWAT

White Adipose Tissue (WAT) exists in various locations in many vertebrates, composed of unilocular adipocytes that store energy in the form of fatty acids, which can be released and broken down into ATP. WAT also performs endocrine functions related to food intake, glucose homeostasis, lipid metabolism, inflammation, and vascular genesis. It is traditionally considered a fat storage region, collectively referred to as subcutaneous fat, near the skin. However, recent advancements in high-resolution microscopy and imaging technologies have revealed a distinct type of WAT storage region located beneath the dermis within skin tissues. Recent research indicates that this type of fat has significantly different anatomical positions and formation processes compared to adjacent subcutaneous fat tissue. These fat cells exhibit rapid transformations in response to various external and internal factors, participating in diverse physiological and pathological processes, including skin-related events such as wound healing, skin fibrosis, regulation of immune responses, and follicular cycling. As a result, these adipose cells have been identified as a unique type of white adipose cells, termed dermal white adipose tissue (dWAT), due to their location in the dermis and their distinctive characteristics in comparison to neighboring subcutaneous fat tissue.

**FIGURE 1:**
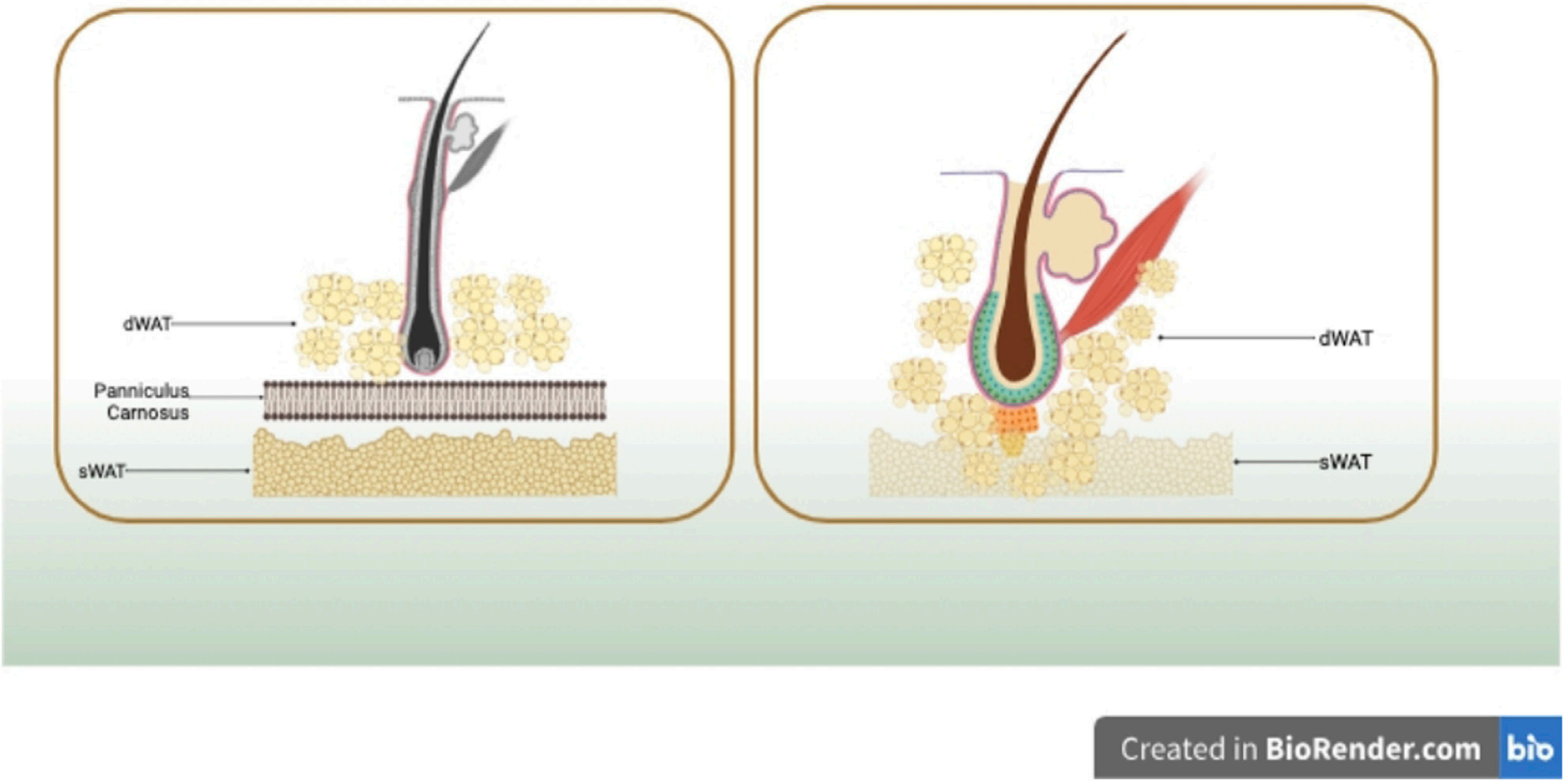
In rodents, sWAT and dWAT are separated by a unique muscle layer known as the meat fascia. In humans, dWAT is concentrated around hair shafts, hair follicles, sebaceous glands, and perilymphatic muscles, forming a distinctive cone-shaped geometric structure. Each dermal cone is composed of two parts, with its upper portion situated within the dermis, while the lower part (referred to as the ‘adipose apex’) spans the dermis and infiltrates into sWAT. Created in BioRender.com

Sbarbati A and Walker GE (Sbarbati et al., 2010a; Walker et al., 2007a), in their anatomical distribution analysis, identified significant differences between dermal white adipose tissue (dWAT) and subcutaneous white adipose tissue (sWAT) based on structural and ultrastructural features. In rodents, sWAT and dWAT are separated by a unique muscle layer known as the meat fascia. sWAT is located in subcutaneous tissues, while dWAT is situated in the reticular dermis layer of the skin. Although many mammals, including humans, lack a distinct meat fascia, small remnants are present in certain regions such as the hands (e.g., palmaris brevis muscle), neck (e.g., platysma muscle), nipples (e.g., subareolar muscle), rectum (e.g., corrugator ani muscle), and scrotum (e.g., dartos muscle) (McMinn, 2003). However, multiple studies (Zhang et al., 2019a; Kruglikov et al., 2019; Smith et al., 2001; Miyazaki et al., 2000; Sbarbati et al., 2010b) indicate the presence of histologically and anatomically distinct adipose tissue layers in the reticular dermis, including species such as pigs and humans, different from sWAT. In pigs, intradermal adipose tissue is divided into three layers, each separated by different fascial layers. Compared to the subcutaneous fat in the lowest layer, the outer and middle layers of fat tissue closer to the dermis in pigs exhibit unique fatty acid compositions, as well as variations in cell count and enzyme activity. In humans, MRI detection reveals that intradermal adipose tissue is divided into two layers. The layer of fat cells closer to the dermis in human skin differs morphologically from the deeper layer of subcutaneous fat cells and secretes more leptin and resistin, showcasing metabolic heterogeneity (Walker et al., 2007a). To further investigate the intradermal adipose layer distinct from sWAT, researchers utilized chemical shift magnetic resonance tomography. The analysis aimed to validate the presence of similar dermal-related fat depots in the human body, revealing their concentration around hair shafts, hair follicles, sebaceous glands, and perilymphatic muscles, forming a unique cone-shaped geometric structure. Each dermal cone is composed of two parts, with its upper portion situated within the dermis, while the lower part (referred to as the ‘adipose apex’) spans the dermis and infiltrates into the sWAT, as illustrated in Figure 1.

### 2.2 Differentiation and formation of dWAT

Moreover, dWAT, in addition to its distinct location from other subcutaneous fat, exhibits specificity in terms of differentiation and formation compared to other subcutaneous tissues. Unlike subcutaneous fat, dWAT undergoes differentiation and formation processes independently of the development of subcutaneous adipocytes, endowing it with unique functions and characteristics.

Studies have indicated that (Wojciechowicz et al., 2013a) dWAT differentiates independently from the development of subcutaneous adipocytes and is generated by cells in the subcutaneous layer of the dermis. Above all Wojciechowicz K’s study (Wojciechowicz et al., 2013a) observed that by embryonic development day 16 (e16), the subcutaneous tissue and the dermal layer began to delineate, signaling the initiation of independent development of dermal white adipose tissue (dWAT) from subcutaneous adipose tissue (sWAT). The two layers of fat separated by the fascia continued to grow independently during subsequent developmental stages. To further confirm that dWAT did not integrate or mix with subcutaneous adipose tissue at any developmental time point, the researchers transplanted dermal tissue isolated from e14.5 mice (before the onset of adipogenesis) under the renal capsule of adult mice. They discovered that dermal adipose tissue developed independently of other tissues and was not influenced by subcutaneous adipose tissue. Thus, it was demonstrated that dWAT originates from cells in the subcutaneous layer of the dermis, developing independently of subcutaneous adipose tissue.

To ascertain that dWAT originates from cells in the subcutaneous layer of the dermis, subsequent research demonstrated that dWAT and dermal fibroblasts are derived from the same lineage. Driskell RR (Driskell et al., 2013a) initially employed transplantation assays and lineage tracing to validate whether the progenitor cells in dWAT are derived from dermal fibroblasts. Skin fibroblasts originate from two distinct lineages—one forming the upper dermis, including the dermal papillae regulating hair growth, and the arrector pili muscle (APM) controlling hair erection. The other lineage forms the lower dermis, including the preadipocytes of subcutaneous tissue. Researchers labeled and located fibroblasts from both the upper and lower dermis, combined them with unlabeled epidermal and dermal cells, and injected them into the chambers implanted in nude/BalbC mice. The Dlk1+Sca1 + fibroblasts (markers for lower dermal fibroblasts) were found to differentiate into preadipocytes but did not differentiate into APM or dermal papillae precursors, generating fibroblasts and preadipocytes. This evidence demonstrates that fibroblasts can differentiate into adipogenic precursors. Therefore, it has been demonstrated that fibroblasts can differentiate into adipogenic precursors. Chia JJ (Chia et al., 2016) further validated this by identifying surface cell markers, confirming that adipogenic precursors present in the reticular dermis of mice originate from mesenchymal fibroblasts. These precursors can undergo directed differentiation into the adipocyte lineage, forming mature dWAT. Consequently, the development of dermal adipose tissue differs from that of subcutaneous adipose tissue, with dWAT established concomitantly with the fibroblast lineage in the dermal interstitium. Another researcher, Rosen, E.D. (Rosen and MacDougald, 2006), using fat markers, demonstrated that the indicators of adipogenesis include the proliferation of preadipocytes, followed by cell cycle arrest and increased expression of PPAR-g and CEBP-a. Differentiated preadipocytes develop cellular mechanisms for lipid synthesis and accumulate lipid droplets. Mature adipocytes, in the end, contain either single-chambered or large lipid droplets and produce specialized hormonally active peptides known as adipokines (Avram MM. et al., 2007). In summary based on the above studies, the formation of dWAT involves two events: the recruitment and proliferation of adipogenic precursor cells and the differentiation and maturation of these precursor cells. Before differentiation, initial mesenchymal stem cells and primary preadipocytes morphologically resemble fibroblasts and can proliferate exponentially. After differentiation initiates, fibroblast-like cells gradually transition into a spherical shape and start accumulating lipids. Eventually, preadipocytes exit the cell cycle, forming dWAT predominantly composed of adipocytes (Tang et al., 2008). In terms of cellular composition, while mature adipocytes constitute the majority of dWAT mass, WAT also includes several other cell types, including immature preadipocyte lineage, blood cells, macrophages, and endothelial cells. Segalla, L. (Segalla et al., 2021a), proposed that dWAT, as a new layer of adipose tissue, also possesses the structural characteristics of adipose tissue, comprising various cell types, including dermal adipocytes (DAs), fibroblast-like adipocyte progenitors, mature adipocytes, dermal fibroblasts (dFBs), macrophages, pericytes, mast cells, endothelial cells, and adipocyte-derived stem cells. In terms of cellular function, the primary structural unit of dWAT is mature DAs, with their cytoplasmic structure and cell nucleus located in a thin layer at the periphery. Besides the well-known roles in lipid storage and release, DAs also play a crucial role in promoting skin immunity, wound healing, and skin fibrosis (Alexander et al., 2015).

However, there is still significant controversy and ongoing debate regarding the differentiation, formation, and cellular composition of dWAT. Further research is needed to clarify these aspects.

## 3 Functions and characteristics of dWAT

The dermal white adipose tissue (dWAT), compared to other fat depots, exhibits multifunctionality. This versatility is evident not only in its ability to differentiate into various cell types but also in its capacity to alter its function and state under different physiological conditions. This plasticity is crucial for the physiological balance and healing processes of the skin, as it enables adaptation to diverse physiological and pathological conditions. dWAT not only participates in wound healing but also plays a significant role in immune and inflammatory responses. Furthermore, dWAT plays a key role in modulating the extracellular matrix (ECM) in the structure and function of the skin. In-depth exploration of the multifunctionality, plasticity, and molecular mechanisms of these cells will contribute to a better understanding of the physiology and disease mechanisms of the skin, providing new hope and possibilities for future therapeutic strategies.

### 3.1 Plasticity and multifunctionality of dWAT

dWAT has unique functions compared to other adipose tissues. Mature dWAT is remarkably plastic, undergoing dedifferentiation and redifferentiation under both physiological and pathological conditions, suggesting that it is capable of altering its state and regaining specific functions.

Researchers have found that under different stimuli such as hair cycling, infection, and wound healing, dWAT can exhibit both volume expansion and reduction. This phenomenon has sparked considerable interest among researchers in the plasticity and multifunctionality of dermal white adipose tissue, leading to extensive studies. Observations indicate that dWAT can differentiate into cells resembling fibroblasts and preadipocytes derived from adipocytes, suggesting a dedifferentiation potential between different states. In order to validate whether dermal adipocytes indeed undergo dedifferentiation, researchers conducted the following experiments. In the second phase of hair growth initiation, Zhang Z (Zhang et al., 2019a) labeled mature dermal adipocytes with GFP and isolated cells from the skin when dWAT exhibited the most significant regression. They used PDGFRα to mark adipocyte precursor cells and preadipocytes. If dermal adipocytes undergo complete dedifferentiation, one would expect to observe double-positive cells for GFP and PDGFRα. Indeed, FACS analysis revealed the presence of a population of CD31^−^CD45-PDGFRα+GFP + cells in the murine dermis following a potent antibiotic pulse. These results were consistent with the dedifferentiation of mature dermal adipocytes into fibroblast-like cells. Subsequently, they purified CD31^−^CD45-PDGFRα+GFP- and CD31^−^CD45-PDGFRα+GFP + cells from the skin for single-cell RNA-Seq. The overall gene expression patterns indicated that dedifferentiated adipocytes largely lost the characteristics of mature adipocytes and regained features resembling complete fibroblasts and preadipocytes. Given Zhang’s discovery that dermal adipocytes can dedifferentiate into fibroblast-like cells, researchers then speculated whether these dedifferentiated adipocytes possess the potential for *in vivo* proliferation or transdifferentiation into other cell types. Subsequently, Marangoni et al. (Marangoni et al., 2015a). employed a bleomycin-induced fibrosis model in mouse skin (Yamamoto et al., 1999). Lineage tracing and *ex vivo* differentiation assays indicated that, when exposed to fibrotic stimuli such as transforming growth factor-beta (TGF-β) or bleomycin, dWAT could undergo direct transdifferentiation into α-smooth muscle actin (α-SMA) positive myofibroblasts, suggesting the potential of adipocytes to transition into myofibroblasts. The results of these experiments provide compelling evidence that dWAT does indeed possess the capability, under specific conditions, to dedifferentiate into various cell types, including fibroblasts and myofibroblasts.

The researchers next verified the possibility that fibroblasts could form adipocytes by transformation or induction: the transformation of dWAT dedifferentiation to fibroblasts appeared to be a reversible process in which myofibroblasts acted as a source of new adipocytes in the wound. Plikus, et al. (Plikus et al., 2017) observed the appearance of new adipocytes within healing wounds, noting that these adipocytes did not form in the non-hairy regions of the wound but specifically developed around new hair follicles. The newly formed adipocytes were capable of producing bone morphogenetic protein (BMP) and reprogramming the surrounding myofibroblasts into adipocytes. In in vitro experiments, when scar-dense cells were treated with BMP4, they were induced to differentiate into lipid-laden adipocytes (Driskell et al., 2013a). To assess the differentiation potential of different dermal fibroblast populations, cells were flow-sorted from P2 PDGFRaH2BeGFP dermis, combined with unlabeled epidermal and dermal cells, and injected into implant sites in nude/BalbC mice. It was found that subcutaneous dermal fibroblasts (Dlk1+Sca1+ and Dlk1-Sca1+) differentiated into adipocytes. Zhang Z (Zhang et al., 2019a) demonstrated in a mouse model that mature dWAT dedifferentiates into smaller fibroblasts during the hair growth phase and redifferentiates back into mature adipocytes during the hair growth initiation phase. These studies collectively provide evidence of the potential mutual conversion between dWAT and fibroblasts. The findings suggest new sources of adipogenic precursor cells and indicate that myofibroblasts can be reprogrammed into adipocytes, offering novel insights into preventing or improving scar formation.

dWAT has the capacity to differentiate into fibroblasts to meet specific tissue repair and functional needs. As widely recognized, fibroblasts play a crucial role in the wound healing process. Through mitosis, they rapidly proliferate and ultimately secrete large amounts of collagen fibers. The regeneration of collagen fibers, along with the formation of new blood vessels, contributes to the development of granulation tissue. Finally, granulation tissue repairs the wound through contraction and aggregation. Therefore, the division of fibroblasts and the production of collagen fibers are key steps in this complex process, ensuring rapid and effective wound healing while minimizing scarring and tissue damage. As a result, the dedifferentiation of dWAT into fibroblasts plays an indispensable role in the wound healing process, aiding in the restoration of the integrity and function of damaged skin. Moreover, fibroblasts derived from the dedifferentiation of adipocytes can undergo redifferentiation into adipocytes, potentially contributing to the reprogramming of disorganized fibrous tissue in scar tissue into adipose tissue, thus improving scar appearance. This physiological process of adipocyte dedifferentiation and redifferentiation repeats during each hair growth cycle, influencing the regulation of the hair growth cycle. This has significant implications for understanding the biological functions of dWAT and its role in the pathological processes of wound healing and scarring.

In summary, these studies reveal the multifunctionality and plasticity of dWAT, as it can differentiate into different cell types, including fibroblasts and myofibroblasts, under different conditions. Based on these findings, the dedifferentiation of dWAT into fibroblasts during skin repair may promote wound healing. Therefore, strategies to intervene in dWAT differentiation may represent a novel approach for intervening in wound healing, treating scars, and addressing fibrosis-related diseases. These discoveries also provide potential targets for future therapeutic strategies, offering the possibility of regulating the differentiation status of dWAT to treat a range of related diseases.

### 3.2 Immune and inflammatory responses

dWAT consists of various immune cells and adipocytes. In addition to the immune cells within dWAT exerting immune effects, adipocytes themselves are also considered to possess immune activity. Moreover, dWAT functions as a regulated lipid layer, providing a defensive role against skin infections.

According to a study conducted in 2020 (Shook et al., 2020a), Nearby adipocytes in the vicinity of skin injuries trigger the release of lipids required for macrophage-mediated inflammation. In the damaged area, dermal white adipose tissue (dWAT) rapidly increases the adipose triglyceride lipase (ATGL)-dependent hydrolysis of triglycerides, releasing saturated and monounsaturated fatty acids to the wound surface. These liberated fatty acids can attract and activate pro-inflammatory Ly6chigh monocyte-derived macrophages, thereby accelerating vascular regeneration in the wound region and promoting the healing of skin wounds. Moreover, the authors achieved the elimination of dWAT lipid breakdown by knocking out the Atgl gene, resulting in reduced lipid content, decreased inflammatory macrophage numbers, and ultimately delayed skin repair in mice. In addition to lipid breakdown, dWAT regulates macrophages’ involvement in tissue repair, regeneration, and immune modulation. When dermal adipocytes exhaust their lipid reserves, they differentiate into Pdgfrα+ Pdgfrβ+ fibroblasts (dFBs), migrate to the injured site, produce extracellular matrix, and further facilitate wound healing. These study results indicate a crucial role for dermal adipocytes in the wound healing process, activating immune cells by releasing fatty acids and enabling mature adipocytes to influence skin inflammation and produce extracellular matrix (ECM) through stromal cells to promote wound recovery. These findings are of paramount significance for a deeper understanding of wound healing mechanisms and the development of relevant therapeutic approaches. In addition to its involvement in immune modulation through lipolysis, dWAT also functions in host defense. Zhang et al. (Zhang et al., 2019a) conducted purification of mature dWAT, comparing the gene expression patterns of purified adipocytes from the subcutaneous layer through RNA sequencing. The study revealed that genes associated with immune response/inflammation were significantly upregulated in dWAT compared to subcutaneous fat, with antimicrobial peptide (Camp) and chemokine (CC motif) ligand 4 (Ccl4) being most abundantly expressed in dermal adipocytes. Conversely, genes related to cell adhesion and migration showed much lower expression levels. Given that the skin serves as the primary antimicrobial barrier, the elevated expression of immune/inflammatory response genes in dermal adipose tissue suggests its crucial role in host defense. This host defense function requires the secretion of antimicrobial peptides by adipocytes, with local changes in adipocyte volume correlating positively with antimicrobial peptide production in response to external stimuli.

In a study by Zhang et al. (Zhang et al., 2015b), it was found that after *Staphylococcus aureus* infection of the skin, preadipocytes rapidly proliferate, dWAT expands rapidly, and antimicrobial peptides are secreted to inhibit bacterial infection. In mice treated with peroxisome proliferator-activated receptor γ inhibitors, leading to impaired dWAT generation, increased susceptibility to infection was observed. Compared to other fat depots, dWAT exhibits a unique expression pattern of Camp. Camp is a crucial antimicrobial peptide that plays a role in physiological processes such as inflammation, infection, wound healing, mast cell chemotaxis, and vascularization, effectively resisting invasive bacterial infections. Further studies have validated these findings, demonstrating that skin infection stimuli can induce an increase in dWAT volume, and dWAT, through the secretion of Camp, prevents mice from being affected by invasive *Staphylococcus aureus* skin infections. Nizet et al. (Avram M. M. et al., 2007) also noted that mice lacking Camp from adipocytes showed decreased defense against *Staphylococcus aureus* infection, correlating with reduced antimicrobial peptide levels. This suggests that the local expansion of dWAT can produce antimicrobial peptides to respond to infections, making it an essential component of the host’s defense against skin infections. Therefore, dWAT is a critical part of the host organism’s defense system, contributing to resistance against pathogens, maintaining physiological balance, and protecting the body from external threats.

In summary, these research findings highlight the crucial role of dWAT in wound healing and immune defense, particularly through its regulation of immune cell activity and antimicrobial peptide expression to maintain skin barrier function. This is of significant importance for a deeper understanding of the functionality of the skin immune system and the potential development of therapeutic approaches.

### 3.3 dWAT and extracellular matrix (ECM)

In addition to mobilizing lipid stores, dWAT regulates the stability of dermal fibroblasts through lipolysis, and its influence on adjacent subcutaneous tissues significantly alters the ECM and mechanical properties of the dermis, contributing to wound stabilization.

The ECM in the dermis is a crucial component that supports and maintains the structure of the skin, primarily synthesized and remodeled by dermal fibroblasts. A study by Zhang et al. (Zhang et al., 2021a). revealed a negative correlation between the content of dWAT and ECM production in dermal fibroblasts. In a mouse model with depleted dWAT, an increase in the expression of genes related to ECM was observed in dermal fibroblasts. Conversely, in mice on a high-fat diet, the expression of ECM-related genes in dermal fibroblasts decreased with an increase in dWAT.

To investigate whether dWAT could influence the metabolism of dermal fibroblasts, experiments were conducted. It was found that dermal fibroblasts are highly sensitive to signals from other cell types, indicating their adaptability to different physiological states and external environments. Upon depletion of dWAT, a downregulation of genes related to fatty acid oxidation was observed in dermal fibroblasts. Co-culturing dermal fibroblasts with conditioned media from dWAT led to a significant upregulation of genes associated with fatty acid oxidation. This suggests that lipids released by dWAT are essential metabolic substrates for neighboring cells, and dWAT, through fatty acid oxidation, regulates the ECM homeostasis in dermal fibroblasts. To further confirm the involvement of fatty acids released by dWAT in the regulation of ECM production in dermal fibroblasts, mice were exposed to a local inhibitor of lipolytic enzyme ATGL. Inhibition of lipolysis led to dermal expansion. Therefore, the regulation by dWAT may contribute to maintaining ECM homeostasis in the dermis. Additionally, Shook BA (Shook et al., 2020a) confirmed that deeper analysis of samples from wound-related areas revealed an abundance of gene clusters associated with myofibroblasts and wound healing-related genes. Four myofibroblast populations enriched in markers related to wound healing and ECM molecules were identified in wound bed samples, suggesting that dWAT differentiates into myofibroblasts in the wound bed after injury.

In summary, dWAT regulates the functions of dermal fibroblasts by releasing fatty acids. This intercellular communication mechanism helps coordinate the functions and maintenance of different skin tissues. Simultaneously, the adjustments made by dWAT to the structure and characteristics of adjacent subcutaneous tissues significantly alter the synthesis and remodeling of ECM and mechanical properties of the dermis, influencing the structure and mechanical properties of the skin, providing a foundation for wound healing. These findings offer crucial insights into the complex interactions among different cell types in the skin and present new directions and possibilities for future research and therapeutic strategies in skin diseases and wound healing.

## 4 dWAT's role in wound healing

dWAT exhibits high plasticity and multifunctionality, playing a crucial role in regulating immune and inflammatory responses, as well as extracellular matrix (ECM) synthesis. These functions and characteristics of dWAT make it pivotal in the wound healing process, contributing significantly to promoting wound closure, regulating immune responses, and modulating skin fibrosis and scarring (Segalla et al., 2021a; Guerrero-Juarez and Plikus, 2018).

### 4.1 dWAT promotes wound healing

The healing of skin wounds is a complex and dynamic process, typically divided into four overlapping stages, including the coagulation phase, inflammatory phase, repair phase, and remodeling phase (Freedman et al., 2023). Following injury, the body initiates the wound healing process, accompanied by the generation of subcutaneous adipose tissue (dWAT), aiming to restore the integrity and composition of the epidermis and dermis to their original structure (Schmidt and Horsley, 2013a).

The mechanism by which dWAT promotes wound healing can be broadly categorized as follows: Firstly, during the early coagulation and inflammatory phases of wound healing, Shook BA demonstrated that dWAT, through the process of β-oxidation or lipolysis, secretes triglycerides to regulate the infiltration of inflammatory macrophages, aiding in initiating the immune response (Shook et al., 2020a). To investigate whether adipocyte lipolysis contributes to the inflammation of skin wounds, the researchers inhibited dermal adipocyte lipolysis. In mice where adipocyte lipolysis was inhibited, the number of Ly6CHi wound bed macrophages decreased by approximately 50% 1.5 days after inhibition, similar to experiments conducted by Ramachandran P (Ramachandran et al., 2012). In the absence of dWAT, wounds in mice lacked the ability to recruit inflammatory Ly6CHi macrophages during the inflammatory phase. Similarly, Shook B (Shook et al., 2016) noted that, in skin wounds lacking dWAT, there was a sustained reduction in the number of macrophages when the local cytokine environment transitioned from pro-inflammatory to anti-inflammatory 3 days after skin injury. These experiments demonstrate that dWAT is essential in this process for effectively engulfing pathogens, resisting pathogenic invasion, and ensuring appropriate immune support to prevent infection. Therefore, dWAT is necessary for activating the inflammatory response and influencing the early stages of skin repair.

In infected wounds, dWAT not only directly combats pathogens by producing antimicrobial peptides (Zhang et al., 2015a) but also mediates immune responses by secreting various bioactive lipid factors and cytokines. Subsequently, as the wound healing progresses into the repair and remodeling phases, dWAT undergoes dedifferentiation and transforms into fibroblast-like cells and myofibroblasts. These cells contribute to building new structures that support wound healing, ensuring the regeneration of tissues at the wound edges (Zhang et al., 2019a). Zhang evaluated the recovery of wounds in FAT-ATTAC mice with adipocyte nutritional deficiencies and their WT (wild-type) counterparts. The regenerative tissues in WT mice were thicker compared to those in FAT-ATTAC mice, and collagen deposition in the wound beds, examined using Masson’s trichrome staining, was lower in FAT-ATTAC mice (Schmidt and Horsley, 2013a). Schmidt BA also confirmed that during the proliferative phase of wound healing on days 5–7, precursor cells of adipocytes proliferate, and adipocytes appear in the wound bed, guiding fibroblasts to migrate into the wound. Functional analysis using adipocyte-deficient.

“lipodystrophic” AZIP/F1 mice suggested that inhibiting adipogenesis impairs fibroblast migration toward the wound center, ultimately leading to long-term loss of skin integrity and wound recurrence (Schmidt and Horsley, 2013a). Furthermore, mature dWAT contains adipose-derived stem cells (ADSCs) that, through various mechanisms such as the secretion of multiple cytokines, regulation of cell signaling pathways, and promotion of cell proliferation, migration, and collagen secretion, contribute to wound healing (Xu et al., 2023). Although the molecular mechanisms of adipocytes in the wound healing process are not fully understood, it can be anticipated that the absence of dWAT may lead to impaired wound healing.

However, in the reconstruction process of skin wound sites, infection is a potential complication that may occur. Current research also indicates that dermal white adipose tissue (dWAT) can produce antimicrobial peptides (AMPs) that directly kill bacteria. When threatened by pathogens such as *Staphylococcus aureus*, local expansion of dWAT occurs, and precursor adipocytes (pADs) rapidly differentiate. During the process of precursor adipocytes transforming into immature adipocytes, they also produce AMPs that directly inhibit pathogen growth, including kallikrein, used to defend against *Staphylococcus* aureus-induced skin infections. However, interestingly, this response appears to decrease with dWAT. Additionally, some studies suggest that Cathelicidin, a type of AMP, may have pro-inflammatory effects, potentially contributing to the chronic low-level inflammation observed in obesity.

Therefore, the Cathelicidins produced by adipocytes may also be involved in the chronic low-level inflammation observed in obesity. These research findings suggest that the local increase in dWAT is an essential host defense response against skin infections. dWAT not only combats skin infections by producing AMPs but also mediates post-injury immune responses by secreting various bioactive lipid factors and cytokines. The response of skin adipocytes to infection may also indirectly enhance immune defense by affecting other processes, such as the oxidative burst of neutrophils, further emphasizing the importance of the subcutaneous adipocyte pool in preventing infections. In summary, dWAT plays a crucial role in defending against skin infections by producing antimicrobial peptides and secreting bioactive lipid factors and cytokines, as well as influencing the oxidative burst response of neutrophils to infected wound sites. These findings contribute to a better understanding of the role of adipocytes in the skin immune system and their potential impact on disease and infection defense.

Moreover, dermal white adipose tissue (dWAT) contains abundant adipose-derived stem cells (ADSCs). Numerous studies suggest that ADSCs can promote wound healing through various mechanisms (Zhao et al., 2018; Xing et al., 2023). Firstly, ADSCs release exosomes that regulate the expression of Bcl-2 and caspase-3 in keratinocytes, inhibiting apoptosis induced by thermal injury. This process alleviates cell G2/M phase arrest, promotes cell cycle progression, and accelerates epithelialization on wound surfaces (Song et al., 2022). It has been demonstrated that injection of cells from the stromal fraction of adipose tissue (Lin- and mesenchymal-derived cells expressing Sca-1, CD29, CD44, CD105) into healing wounds has been shown to enhance keratinocyte migration and inhibit apoptosis to accelerate wound closure, promote cutaneous wound healing, and reduce scar formation (Jackson et al., 2012a). Furthermore, in response to tissue damage, ADSCs mitigate oxidative stress-induced damage to endothelial cells by regulating HIF1α. This regulation enhances endothelial cell proliferation, migration, and angiogenesis. Adipose-derived stem cells almost secrete all growth factors associated with normal wound healing, such as vascular endothelial growth factor (VEGF) and epidermal growth factor (EGF). Additionally, they can further increase the release of these growth factors in hypoxic conditions. The paracrine effects of ADSCs, including the secretion of KGF-1 and PDGF-BB, mediate increased reepithelialization and vascular density in skin wounds (Alexaki et al., 2012; Huang et al., 2012). Moreover, ADSCs alleviate excessive inflammatory responses and oxidative stress during tissue damage by polarizing macrophages towards the pro-repair M2 phenotype. Transplanting adipose derived stem cells into wound beds can change the phenotype of wound bed macrophages, induce TGF-β(1)-dependent angiogenesis, fibroblast differentiation, and granulation tissue formation, enhancing overall tissue repair. Additionally, under various stimuli and induction factors, ADSCs can differentiate into adipocytes, actively participating in the wound healing process (Jackson et al., 2012b). These characteristics enable ADSCs to play an active role in promoting wound healing through both their differentiation capacity and paracrine functions.

**FIGURE 2:**
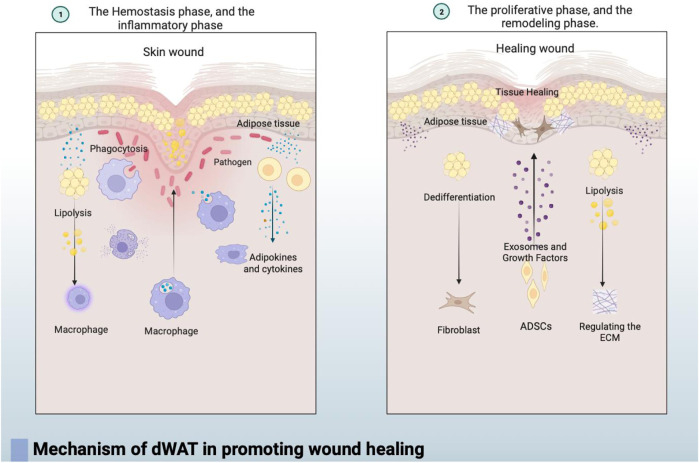
① In the early stages of wound healing, during the coagulation and inflammation phases, dWAT regulates the infiltration of inflammatory macrophages through fat breakdown, contributing to the initiation of the immune response. dWAT also mediates the immune response by secreting various bioactive lipid factors and cytokines.② As wound healing progresses into the repair and remodeling phases, dWAT undergoes dedifferentiation and transforms into fibroblast-like cells. Simultaneously, dWAT regulates the wound’s surrounding extracellular matrix (ECM) through lipolysis, significantly altering the dermis’s ECM and mechanical properties to stabilize the wound. Additionally, adipose-derived stem cells (ADSCs) in dWAT contribute to wound healing by secreting exosomes and various cytokines, regulating cell signaling to enhance cell proliferation, migration, and collagen secretion.

In summary, the mechanism by which dermal white adipose tissue (dWAT) promotes wound healing can be roughly divided into the following three points: the breakdown of adipocytes in the dermal fat layer can promote wound healing by regulating the infiltration of inflammatory macrophages. Additionally, the loss of dermal adipocytes occurs at the wound edge, and cells derived from adipocytes become wound bed myofibroblasts that produce extracellular matrix (ECM) during the proliferative phase of repair. Secondly, dWAT plays a crucial role in the defense against infected wounds, not only by directly combating pathogens through the production of antimicrobial peptides such as cathelicidins but also by mediating the immune response through the secretion of various bioactive lipid factors and cytokines. Thirdly, adipose-derived stem cells (ADSCs) in mature dWAT contribute to wound healing by secreting various cytokines, regulating cell signaling pathways, and facilitating cell proliferation, migration, and collagen secretion through multiple mechanisms. In conclusion, dWAT plays a vital role in initiating the immune response and promoting tissue regeneration and repair during the process of skin wound healing. Its importance throughout different stages of functional regulation influences the final healing outcome, emphasizing its significance in the skin healing process and providing valuable clues for further research into the mechanisms of wound healing (In Figure 2).

### 4.2 Regulation of immune response by dWAT in wound healing

The skin immune system is a crucial defense mechanism that protects the human body from external pathogens and harmful substances. Dermal white adipose tissue (dWAT), a regulated lipid layer, plays a fundamental role in maintaining the integrity of the skin barrier and is involved in the skin’s immune defense system (Belkaid and Segre, 2014; Celebi Sözener et al., 2020).

dWAT not only contains nearly all types of immune cells but also its primary constituent, adipocytes, plays a role in immune response and regulation (Chen et al., 2019). Therefore, dWAT primarily functions in the complex immune network by participating in pathogen engulfment and tissue repair and regeneration (Frasca and Blomberg, 2020a). In conclusion, dWAT plays a crucial role in the skin’s immune defense system by contributing to the complex immune network, engaging in pathogen engulfment, and facilitating tissue repair and regeneration (Frasca and Blomberg, 2020a).

dWAT is involved in the immune regulation of the skin, with its immune cells primarily composed of leukocytes. Research by Brüggen et al. (Brüggen et al., 2019) investigated the quality, quantity, and distribution of leukocytes in dWAT, comparing them to leukocytes in the skin. Fat tissue was extracted from the inner thigh and skin specimens of healthy young women, and experiments were conducted on both cell suspensions and tissue sections. Leukocytes isolated from dWAT exhibited significant differences in quality compared to those separated from the epidermis and dermal layers. The proportion of macrophages in dWAT was significantly higher than in skin tissue, with M2-type macrophages predominating, characterized by the expression of CD163 and the absence of CD11c. The second-largest leukocyte subset in dWAT was T lymphocytes. To characterize the T cells in dWAT, researchers assessed the expression of their transcription factors in tissue sections, revealing the presence of transcription factors for helper T cell types 1 and 2. Further examination of the innate counterparts of T cells, known as innate lymphoid cells (ILCs), showed that natural killer cells constituted approximately 0.75% and helper ILCs accounted for about 0.02%. Among dWAT ILCs, ILC2 (CD45^+^ cells, CD127+ CD161+ CRTH2+ cells) was identified as the most prominent subset, averaging at 69.3%.In summary, dWAT harbors a distinct leukocyte compartment, with a higher proportion of M2-type macrophages and a significant presence of T lymphocytes, particularly of the helper T cell types 1 and 2. Additionally, innate lymphoid cells, particularly ILC2, play a notable role in the leukocyte composition of dWAT.

Furthermore, the adipocytes, the primary components of dWAT, play a crucial role in controlling inflammation and regulating immune responses. Firstly, dWAT is involved in host defense against various pathogenic microorganisms by expressing Toll-like receptors (TLRs), which are essential pattern recognition receptors contributing to the host’s defense against pathogens (Caër et al., 2017a; Miller and Modlin, 2007). Adipocytes participate in both innate and adaptive immune responses by producing cytokines, chemokines, antimicrobial peptides, co-stimulatory, and adhesion molecules. Moreover, adipocytes can produce chemokine receptors such as IL-1R1, IL-17RA, TNF-R1, and IL-10Rα, triggering downstream inflammatory signals (Caër et al., 2017aRajbhandari et al., 2018). Additionally, adipocytes can secrete molecules known as adipokines, such as inflammatory (IL-1β and IL-17)

and fibrotic (TGF-β1) factors. For example, the adipokine leptin can promote the production of pro-inflammatory cytokines and activate CD4^+^ and CD8^+^ T cells [93] (Procaccini et al., 2013). In contrast, the adipokine adiponectin exerts an anti-inflammatory effect by inhibiting tumor necrosis factor-alpha expression in monocytes, suppressing macrophage and T cell proliferation, and inhibiting the production of IL-10 [94] (Kawai et al., 2021).

Furthermore, dWAT secretes molecules such as plasminogen activator inhibitor-1 (PAI-1), MCP-1, interleukin-8 (IL-8), and IL-6, at least as much as leptin. In a mouse endocarditis model, leptin levels significantly increased, while adiponectin levels markedly decreased, emphasizing the importance of adipocytes in immune regulation and inflammatory responses within dWAT (Schmid et al., 2017). These findings underscore the significance of adipocytes in dWAT in immune modulation and inflammation responses, highlighting their diverse functions in the skin’s immune system.

dWAT performs a crucial role in the immunological system of the skin, encompassing various types of immune cells, with M2-type macrophages and Th2/Treg cells predominantly present. These immune cells possess regulatory functions, capable of suppressing the inflammatory processes in the superficial layers of the skin, while promoting vascular regeneration and tissue repair. Adipocytes constitute the primary components of dWAT, actively participating in immune regulation and inflammatory responses through the expression of various receptors and molecules, as well as the production of diverse adipokines.

### 4.3 dWAT’s involvement in scar formation during wound healing

dWAT plays a role in the wound healing process, and pathological scar formation and fibrosis are abnormal outcomes of wound healing. It can be hypothesized that there is a connection between dWAT and scar formation and fibrosis. A decrease in the content of dWAT in the skin is typically associated with excessive proliferation and transdifferentiation of fibroblasts and myofibroblasts, as well as the excessive deposition of extracellular matrix, leading to scar formation.

There are structural and content differences in dWAT in different body regions, suggesting a regional correlation between scar formation and dWAT (Ma et al., 2023). Histological analysis of human skin samples reveals that dWAT is primarily present in regions prone to hypertrophic scars, such as the face, neck, chest, abdomen, and back, while it is less distributed in areas with low scar formation potential, such as the palms, early fetal skin, and scalp. Additionally, in animals that are less prone to scar formation after wound healing, such as rats and rabbits, there is a lower quantity of dWAT. This suggests that the content of dWAT may contribute to scar formation and fibrosis in humans and certain animals (Kruglikov and Scherer, 2016b). Therefore, the regional differences between scars and dWAT imply that the structure and content of dWAT vary in different body regions, reflecting variability in wound healing and skin fibrosis in these areas.

The mechanism of the effect of dWAT on scarring and fibrosis has also been explored by researchers. Marangoni RG (Marangoni et al., 2015b) suggests that the loss of adipose tissue and the transformation of adipocytes into myofibroblasts may be primary factors in the pathogenesis of skin fibrosis. The loss of adipose tissue and the transformation of adipocytes into myofibroblasts may be primary factors in the pathogenesis of skin fibrosis. In mice induced with fibrosis using bleomycin, Marangoni observed the replacement of subcutaneous adipose tissue with fibrous tissue. The expression of the typical adipogenic marker PPARγ in the skin decreased, preceding the expression of dermal fibrosis markers. This demonstrates that the content of dWAT significantly influences the process of skin fibrosis. Furthermore, dWAT contains matrix cells derived from reparative fat and expresses anti-fibrotic cytokines such as adiponectin. Adiponectin, primarily secreted by adipocytes, exerts its anti-fibrotic effects by activating the transmembrane receptors AdipoR1 and AdipoR2, and initiating the adenosine monophosphate (AMP) signaling pathway (Zhang et al., 2021a). Serum levels of adiponectin in patients with hypertrophic scars were found to be lower compared to normal individuals. In a systemic sclerosis mouse model, adiponectin attenuated the activation of fibroblasts, indicating that the absence of adiponectin increases signaling transduction, exacerbating skin fibrosis (Wang et al., 2017). As dWAT is lost, the protective mechanisms against fibrosis in the skin are diminished, further contributing to skin fibrosis and damage. Marangoni RG’s cell fate mapping study, conducted in mice using a transgenic construct with Cre recombinase driven by the adiponectin promoter, revealed that adiponectin-positive progenitor cells lacking intradermal adipose tissue compartments gradually lost adipocyte markers over time. These observations establish a new link between the loss of intradermal adipose tissue and dermal fibrosis. Additional *in vitro* studies support these conclusions, indicating that adipose-derived stem cells (ADSCs) can differentiate into myofibroblasts or fibroblast-like cells using growth factors present in the wound bed. In this context, transforming growth factor-beta (TGF-β) can stimulate the myofibroblast phenotype (Marangoni et al., 2015c). After culturing ADSCs in media for 10 days, Marangoni RG induced the ADSCs with TGF-β, resulting in the generation of myofibroblasts expressing both peripin and α-SMA typical characteristics, while completely losing the adipocyte markers.

It is noteworthy that Yun IS (Yun et al., 2012) also demonstrated that myofibroblasts and fibroblast-like cells can redifferentiate into adipocytes. This finding suggests an important mechanism indicating the bidirectional differentiation capability between adipocytes and myofibroblasts. Current clinical studies have found that autologous dWAT transplantation can significantly improve surface scars, making hypertrophic scars softer and the texture closer to normal tissue (Bruno et al., 2013a). Histological results show that dWAT transplantation stimulates the regeneration of elastic fibers in scars, promoting the restoration of orderly arranged and shaped collagen fibers from their chaotic and disordered state. This indicates that autologous dWAT transplantation has a potent collagen remodeling function and is an effective method for treating hypertrophic scars (Bruno et al., 2013a). These findings underscore the potential applications of dWAT and fat grafting in the treatment of skin fibrosis and hypertrophic scars.

Additionally, adipose-derived stem cells (ADSCs) exhibit anti-fibrotic effects. Hypertrophic scars are typically characterized by abnormal extracellular matrix (ECM), excessive collagen deposition, and abnormal arrangement. ADSCs can inhibit scar fibrosis by downregulating the TGF-β1/Smad signaling pathway (Borovikova et al., 2018) Zhang Q (Zhang Q. et al., 2015) confirmed that local injection of ADSCs significantly reduces the levels of type I, type III collagen, TGF-β1, and α-smooth muscle actin in hypertrophic scar tissues in rabbit ears. Further research indicates that after ADSC injection, the expression of decorin (DCN), an antagonistic factor against TGF-β1, increases. DCN effectively inhibits fibroblast contraction and collagen synthesis, thereby improving scars (Chu et al., 2018). The application of ADSCs can indeed regulate the early stages of scar formation and remodeling, and the improvement in scar formation is associated with the inhibition of TGF-β.

Therefore, the loss of dWAT is associated with scar formation and fibrosis, where mature adipocytes can transdifferentiate into fibroblasts, leading to fibroproliferation and skin fibrosis. The structural and content differences of dWAT in different body regions may influence scar formation, suggesting a regional correlation of dWAT in wound healing and skin fibrosis. The application of ADSCs can regulate scar formation and remodeling, while autologous dWAT transplantation demonstrates significant therapeutic effects, making scars softer and promoting the regeneration of elastic fibers. Currently, researchers are considering whether it is possible to reprogram adipocytes into myofibroblasts, preventing or reversing the transdifferentiation of adipocytes into myofibroblasts. Enhancing the survival of reparative adipose derived stem cells (ADSCs) and the expression of anti-fibrotic cytokines could be potential therapeutic approaches for effective scar and fibrosis treatment.

## 5 Prospects

Until recently, the dWAT layer has received minimal attention. We are now increasingly recognizing the high adaptability of these fat cells, and variations in dWAT under different physiological and pathological conditions may hold significant implications for various processes.

dWAT, owing to its high plasticity and multifunctionality, plays a substantial role in regulating immune-inflammatory responses and extracellular matrix (ECM) synthesis. It holds crucial clinical value in wound healing by promoting wound repair, regulating immune responses, and inhibiting scar formation and fibrosis. Additionally, dWAT plays a broad and crucial regulatory role in the pathophysiology of the skin. Intervention in dWAT has shown preliminary effectiveness in promoting wound healing, reducing scar proliferation, and stimulating hair regeneration in clinical treatments. Adipose-derived stem cells (ADSCs), present in dWAT, exhibit advantages such as widespread sourcing, strong amplification capabilities, and stable induced differentiation. In studies addressing tissue injuries and various diseases, ADSCs have demonstrated positive therapeutic effects. Early international research and clinical applications of ADSCs have achieved breakthroughs in tissue engineering technologies based on ADSCs, successfully reconstructing tissues like bone, cartilage, and blood vessels. This lays a significant foundation for the clinical application of dWAT in skin aging, wound healing, scar prevention and treatment, and hair regeneration.

There are numerous aspects of dWAT’s role in skin physiology and potential application areas that merit in-depth research. Understanding how factors influence the development and function of dWAT can contribute to a better comprehension of complex mechanisms in skin physiology. Future research can explore manipulating the plasticity of dermal fat cells to promote wound healing, reduce scar proliferation, and stimulate hair regeneration. Additionally, investigating the effective antibacterial role of dWAT in infected wounds is crucial. Future clinical studies may further explore the potential of dWAT in treating skin diseases such as psoriasis, scleroderma, alopecia, and atopic dermatitis. Studies can also examine whether dWAT thickness is genetically determined and the impact of gene-environment interactions on disease onset. This aids in accurately predicting patient risks and developing personalized treatment plans. Further research into the molecular mechanisms between dWAT and diseases, such as fibrosis and cancer, can reveal new therapeutic targets and strategies. Understanding these mechanisms will contribute to the development of more effective treatment methods.

In conclusion, dWAT plays multiple roles in skin physiology, and future research will help unveil more of its mysteries and potential applications, providing more opportunities for skin health and disease treatment. By delving deeper into the functions and interrelationships of dWAT, we can gain a better understanding of skin biology, offering insights and innovations for future clinical practices.

## References

[B1] Alexaki V. I. Simantiraki D. Panayiotopoulou M. Rasouli O. Venihaki M. Castana O. (2012). Adipose tissue-derived mesenchymal cells support skin reepithelialization through secretion of KGF-1 and PDGF-BB: comparison with dermal fibroblasts. Cell. Transpl. 21 (11), 2441–2454. 10.3727/096368912X637064 22507764

[B2] Alexander C. M. Kasza I. Yen C. L. E. Reeder S. B. Hernando D. Gallo R. L. (2015). Dermal white adipose tissue: a new component of the thermogenic response. J. Lipid Res. 56 (11), 2061–2069. 10.1194/jlr.R062893 26405076 PMC4617393

[B3] Avram M. M. Avram A. S. James W. D. (2007a). Subcutaneous fat in normal and diseased states. 3. Adipogenesis: from stem cell to fat cell. J. Am. Acad. Dermatol 56, 472–492. 10.1016/j.jaad.2006.06.022 17317490

[B4] Avram M. M. Avram A. S. James W. D. (2007b). Subcutaneous fat in normal and diseased states 3. Adipogenesis: from stem cell to fat cell. J. Am. Acad. Dermatol 56 (3), 472–492. 10.1016/j.jaad.2006.06.022 17317490

[B5] Belkaid Y. Segre J. A. (2014). Dialogue between skin microbiota and immunity. Science 346 (6212), 954–959. 10.1126/science.1260144 25414304

[B6] Borovikova A. A. Ziegler M. E. Banyard D. A. Wirth G. A. Paydar K. Z. Evans G. R. D. (2018). Adipose-derived tissue in the treatment of dermal fibrosis: antifibrotic effects of adipose-derived stem cells. Ann. Plast. Surg. 80 (3), 297–307. 10.1097/SAP.0000000000001278 29309331

[B7] Brüggen M. C. Strobl J. Koszik F. Naito R. Vierhapper M. (2019). Subcutaneous white adipose tissue of healthy young individuals harbors a leukocyte compartment distinct from skin and blood. J. Investig. Dermatol 139 (9), 2052–2055. 10.1016/j.jid.2019.02.034 30974167

[B8] Bruno A. Delli Santi G. Fasciani L. Cempanari M. Palombo M. Palombo P. (2013a). Burn scar lipofilling: immunohistochemical and clinical outcomes. J. Craniofac Surg. 24 (5), 1806–1814. 10.1097/SCS.0b013e3182a148b9 24036785

[B9] Caër C. Rouault C. Le Roy T. Poitou C. Aron-Wisnewsky J. Torcivia A. (2017a). Immune cell-derived cytokines contribute to obesity-related inflammation, fibrogenesis and metabolic deregulation in human adipose tissue. Sci. Rep. 7 (1), 3000. 10.1038/s41598-017-02660-w 28592801 PMC5462798

[B10] Celebi Sözener Z. Cevhertas L. Nadeau K. Akdis M. Akdis C. A. (2020). Environmental factors in epithelial barrier dysfunction. J. Allergy Clin. Immunol. 145 (6), 1517–1528. 10.1016/j.jaci.2020.04.024 32507229

[B11] Chen S. X. Zhang L. J. Gallo R. L. (2019). Dermal white adipose tissue: a newly recognized layer of skin innate defense. J. Investig. Dermatol 139 (5), 1002–1009. 10.1016/j.jid.2018.12.031 30879642

[B12] Chia J. J. Zhu T. Chyou S. Dasoveanu D. C. Carballo C. Tian S. (2016). Dendritic cells maintain dermal adiposeederived stromal cells in skin fibrosis. J. Clin. Investig. 126, 4331e45. 10.1172/JCI85740 27721238 PMC5096920

[B13] Chu H. Wang Y. Wang X. Song X. Liu H. Li X. (2018). Effects of transplanted adipose derived stem cells on the expressions of α-SMA and DCN in fibroblasts of hypertrophic scar tissues in rabbit ears. Exp. Ther. Med. 16 (3), 1729–1734. 10.3892/etm.2018.6383 30186394 PMC6122172

[B14] Driskell R. R. Lichtenberger B. M. Hoste E. Kretzschmar K. Simons L. Charalambous F. (2013a). Distinct fi broblast lineages deter-mine dermal architecture in skin development and repair. Nature 504, 277–281. 10.1038/nature12783 24336287 PMC3868929

[B15] Frasca D. Blomberg B. B. (2020a). Adipose tissue: a tertiary lymphoid organ: does it change with age? Gerontology 66 (2), 114–121. 10.1159/000502036 31412335 PMC7018534

[B16] Freedman B. R. Hwang C. Talbot S. Hibler B. Matoori S. Mooney D. J. (2023). Breakthrough treatments for accelerated wound healing. Sci. Adv. 9 (20), eade7007. 10.1126/sciadv.ade7007 37196080 PMC10191440

[B17] Guerrero-Juarez C. F. Plikus M. V. (2018). Emerging nonmetabolic functions of skin fat. Nat. Rev. Endocrinol. 14 (3), 163–173. 10.1038/nrendo.2017.162 29327704 PMC6042872

[B18] Huang S. P. Hsu C. C. Chang S. C. Wang C. H. Deng S. C. Dai N. T. (2012). Adipose-derived stem cells seeded on acellular dermal matrix grafts enhance wound healing in a murine model of a full-thickness defect. Ann. Plast. Surg. 69 (6), 656–662. 10.1097/SAP.0b013e318273f909 23154338

[B19] Jackson W. M. Nesti L. J. Tuan R. S. (2012a). Concise review: clinical translation of wound healing therapies based on mesenchymal stem cells. Stem Cells Transl. Med. 1 (1), 44–50. 10.5966/sctm.2011-0024 23197639 PMC3727688

[B20] Jackson W. M. Nesti L. J. Tuan R. S. (2012b). Concise review: clinical translation of wound healing therapies based on mesenchymal stem cells. Stem Cells Transl. Med. 1 (1), 44–50. 10.5966/sctm.2011-0024 23197639 PMC3727688

[B21] Kasza I. Hernando D. Roldán-Alzate A. Alexander C. M. Reeder S. B. (2016). Thermogenic profiling using magnetic resonance imaging of dermal and other adipose tissues. JCI Insight 1 (13), e87146. 10.1172/jci.insight.87146 27668285 PMC5034877

[B22] Kasza I. Suh Y. Wollny D. Clark R. J. Roopra A. Colman R. J. (2014). Syndecan-1 is required to maintain intradermal fat and prevent cold stress. PLoS Genet. 10 (8), e1004514. 10.1371/journal.pgen.1004514 25101993 PMC4125098

[B23] Kawai T. Autieri M. V. Scalia R. (2021). Adipose tissue inflammation and metabolic dysfunction in obesity. Am. J. Physiol. Cell. Physiol. 320 (3), C375–C391. 10.1152/ajpcell.00379.2020 33356944 PMC8294624

[B24] Kruglikov I. L. Scherer P. E. (2016). Dermal adipocytes and hair cycling: is spatial heterogeneity a characteristic feature of the dermal adipose tissue depot? Exp. Dermatol 25 (4), 258–262. 10.1111/exd.12941 26781768 PMC4805479

[B25] Kruglikov I. L. Scherer P. E. (2016b). Dermal adipocytes: from irrelevance to metabolic targets? Trends Endocrinol. Metab. 27, 1–10. 10.1016/j.tem.2015.11.002 26643658 PMC4698208

[B26] Kruglikov I. L. Zhang Z. Scherer P. E. (2019). The role of immature and mature adipocytes in hair cycling. Trends Endocrinol. Metab. 30, 93–105. 10.1016/j.tem.2018.11.004 30558832 PMC6348020

[B27] Ma Y. Liu Z. Miao L. Jiang X. Ruan H. Xuan R. (2023). Mechanisms underlying pathological scarring by fibroblasts during wound healing. Int. Wound J. 20, 2190–2206. 10.1111/iwj.14097 36726192 PMC10333014

[B28] Marangoni R. G. Korman B. D. Wei J. Scherer E. Tourtellotte W. G. Varga J. (2015a). Myofibroblasts in murine cutaneous fibrosis originate from adiponectin-positive intradermal progenitors. Arthritis Rheumatol. 67, 1062–1073. 10.1002/art.38990 25504959 PMC4472310

[B29] Marangoni R. G. Korman B. D. Wei J. Wood T. A. Graham L. V. Whitfield M. L. (2015b). Myofibroblasts in murine cutaneous fibrosis originate from adiponectin-positive intradermal progenitors. Arthritis Rheumatol. 67, 1062–1073. 10.1002/art.38990 25504959 PMC4472310

[B30] Marangoni R. G. Korman B. D. Wei J. Wood T. A. Graham L. V. Whitfield M. L. (2015c). Myofibroblasts in murine cutaneous fibrosis originate from adiponectin-positive intradermal progenitors. Arthritis Rheumatol. 67 (4), 1062–1073. 10.1002/art.38990 25504959 PMC4472310

[B31] McMinn R. M. H. (2003). “Introduction to regional anatomy,” in McMinn RMH. Last’s anatomy—regional and applied. 9th ed. (Marrickville, NSW, Australia: Churchill Livingstone), 3.

[B32] Miller L. S. Modlin R. L. (2007). Toll-like receptors in the skin. Semin. Immunopathol. 29 (1), 15–26. 10.1007/s00281-007-0061-8 17621951

[B33] Miyazaki M. Kim Y. C. Gray-Keller M. P. Attie A. D. Ntambi J. M. (2000). The biosynthesis of hepatic cholesterol esters and triglycerides is impaired in mice with a disruption of the gene for stearoyl-CoA desaturase 1. J. Biol. Chem. 275 (39), 30132–30138. 10.1074/jbc.M005488200 10899171

[B34] Plikus M. V. Guerrero-Juarez C. F. Ito M. Li Y. R. Dedhia P. H. Zheng Y. (2017). Regeneration of fat cells from myofibroblasts during wound healing. Sci- ence 355 (6326), 748–752. 10.1126/science.aai8792 PMC5464786 28059714

[B35] Procaccini C. De Rosa V. Galgani M. Carbone F. La Rocca C. Formisano L. (2013). Role of adipokines signaling in the modulation of T cells function. Front. Immunol. 4, 332–341. 10.3389/fimmu.2013.00332 24151494 PMC3799205

[B36] Querleux B. Cornillon C. Jolivet O. Bittoun J. (2002). Anatomy and physiology of subcutaneous adipose tissue by *in vivo* magnetic resonance imaging and spectroscopy: relationships with sex and presence of cellulite. Skin. Res. Technol. 8 (2), 118–124. 10.1034/j.1600-0846.2002.00331.x 12060477

[B37] Rajbhandari P. Thomas B. J. Feng A. C. Hong C. Wang J. Vergnes L. (2018). IL-10 signaling remodels adipose chromatin architecture to limit thermogenesis and energy expenditure limit thermogenesis and energy expenditure. Cell. 172 (1-2), 218–233.e17. 10.1016/j.cell.2017.11.019 29249357 PMC5766418

[B38] Ramachandran P. Pellicoro A. Vernon M. A. Boulter L. Aucott R. L. Ali A. (2012). Differential Ly-6C expression identifies the recruited macrophage phenotype, which orchestrates the regression of murine liver fibrosis. Proc. Natl. Acad. Sci. U. S. A. 109 (46), E3186–E3195. 10.1073/pnas.1119964109 23100531 PMC3503234

[B39] Rosen E. D. MacDougald O. A. (2006). Adipocyte differentiation from the inside out. Nat. Rev. Mol. Cell. Biol. 7 (12), 885–896. 10.1038/nrm2066 17139329

[B40] Sbarbati A. Accorsi D. Benati D. Marchetti L. Orsini G. Rigotti G. (2010a). Subcutaneous adipose tissue classification. Eur. J. Histochem 54 (4), e48. 10.4081/ejh.2010.e48 21263747 PMC3167328

[B41] Sbarbati A. Accorsi D. Benati D. Marchetti L. Orsini G. Rigotti G. (2010b). Subcutaneous adipose tissue classification. Eur. J. Histochem 54 (4), e48. 10.4081/ejh.2010.e48 21263747 PMC3167328

[B42] Schmid A. Karrasch T. Thomalla M. Schlegel J. Salzberger B. Schäffler A. (2017). Innate immunity of adipose tissue in rodent models of local and systemic *Staphylococcus aureus* infection. Mediat. Inflamm. 2017, 5315602. 10.1155/2017/5315602 PMC5385907 28428684

[B43] Schmidt B. A. Horsley V. (2013a). Intradermal adipocytes mediate fibroblast recruitment during skin wound healing. Development 140 (7), 1517–1527. 10.1242/dev.087593 23482487 PMC3596993

[B44] Segalla L. Chirumbolo S. Sbarbati A. (2021a). Dermal white adipose tissue: much more than a metabolic, lipid-storage organ? Tissue Cell. 71, 101583. 10.1016/j.tice.2021.101583 34171520

[B45] Shook B. Xiao E. Kumamoto Y. Iwasaki A. Horsley V. (2016). CD301b+ macrophages are essential for effective skin wound healing. J. Investig. Dermatol 136 (9), 1885–1891. 10.1016/j.jid.2016.05.107 27287183 PMC5727894

[B46] Shook B. A. Wasko R. R. Mano O. Rutenberg-Schoenberg M. Rudolph M. C. Zirak B. (2020a). Dermal adipocyte lipolysis and myofibroblast conversion are required for efficient skin repair. Cell. Stem Cell. 26 (6), 880–895. 10.1016/j.stem.2020.03.013 32302523 PMC7853423

[B47] Smith S. R. Lovejoy J. C. Greenway F. Ryan D. deJonge L. de la Bretonne J. (2001). Contributions of total body fat, abdominal subcutaneous adipose tissue compartments, and visceral adipose tissue to the metabolic complications of obesity. Metabolism 50 (4), 425–435. 10.1053/meta.2001.21693 11288037

[B48] Song M. Bai X. Wang D. Wang Q. Pan L. He P. (2022). Combined application of moist exposed burn ointment and maggot therapy in wound healing. J. Wound Care 31 (Suppl. 10), S41–s52. 10.12968/jowc.2022.31.Sup10.S41 36240870

[B49] Stepp M. A. Gibson H. E. Gala P. H. Iglesia D. D. S. Pajoohesh-Ganji A. Pal-Ghosh S. (2002). Defects in keratinocyte activation during wound healing in the syndecan-1-deficient mouse. J. Cell. Sci. 115 (Pt 23), 4517–4531. 10.1242/jcs.00128 12414997

[B50] Tang W. Zeve D. Suh J. M. Bosnakovski D. Kyba M. Hammer R. E. (2008). White fat progenitor cells reside in the adipose vasculature. Science 322 (5901), 583–586. 10.1126/science.1156232 18801968 PMC2597101

[B51] Walker G. E. Verti B. Marzullo P. Savia G. Mencarelli M. Zurleni F. (2007a). Deep subcutaneous adipose tissue: a distinct abdominal adipose depot. Obes. (Silver Spring) 15 (8), 1933–1943. 10.1038/oby.2007.231 17712110

[B52] Wang Y. Liang B. Lau W. B. Du Y. Guo R. Yan Z. (2017). Restoring diabetes-induced autophagic flux arrest in ischemic/reperfused heart by ADIPOR (adiponectin receptor) activation involves both AMPK-dependent and AMPK-independent signaling. Autophagy 13 (11), 1855–1869. 10.1080/15548627.2017.1358848 28825851 PMC5788496

[B53] Wojciechowicz K. Gledhill K. Ambler C. A. Manning C. B. Jahoda C. A. (2013a). Development of the mouse dermal adipose layer occurs independently of subcutaneous adipose tissue and is marked by restricted early expression of FABP4. PLoS One 8 (3), e59811. 10.1371/journal.pone.0059811 23555789 PMC3608551

[B54] Xing N. Huo R. Wang H. T. Yang J. C. Chen J. Peng L. (2023). Research advances of adipose stem cell matrix gel in promoting wound healing. Zhonghua Shao Shang Za Zhi 39 (1), 81–84. 10.3760/cma.j.cn501120-20211204-00404 PMC11630351 36740431

[B55] Xu Z. H. Ma M. H. Li Y. Q. Li L. L. Liu G. H. (2023). Progress and expectation of stem cell therapy for diabetic wound healing. World J. Clin. Cases 11 (3), 506–513. 10.12998/wjcc.v11.i3.506 36793646 PMC9923865

[B56] Yamamoto T. Takagawa S. Katayama I. Yamazaki K. Hamazaki Y. Shinkai H. (1999). Animal model of sclerotic skin. I: local injections of bleomycin induce sclerotic skin mimicking scleroderma. J. Investig. Dermatol 112 (4), 456–462. 10.1046/j.1523-1747.1999.00528.x 10201529

[B57] Yun I. S. Jeon Y. R. Lee W. J. Lee J. W. Rah D. K. Tark K. C. (2012). Effect of human adipose derived stem cells on scar formation and remodeling in a pig model: a pilot study. Dermatol Surg. 38 (10), 1678–1688. 10.1111/j.1524-4725.2012.02495.x 22804839

[B58] Zhang L. J. Guerrero-Juarez C. F. Hata T. Bapat S. P. Ramos R. Plikus M. V. (2015a). Innate immunity. Dermal adipocytes protect against invasive *Staphylococcus aureus* skin infection. Science 347 (6217), 67–71. 10.1126/science.1260972 25554785 PMC4318537

[B59] Zhang L. J. Guerrero-Juarez C. F. Hata T. Bapat S. P. Ramos R. Plikus M. V. (2015c). Innate immunity. Dermal adipocytes protect against invasive *Staphylococcus aureus* skin infection. Science 347, 67–71. 10.1126/science.1260972 25554785 PMC4318537

[B60] Zhang L. J. Guerrero-Juarez F. Hata T. Plikus M. V. Gallo R. L. (2015b). Innate immunity. Dermal adi-pocytes protect against invasive *Staphylococcus aureus* skin infec-tion. Science 347, 67–71. 10.1126/science.1260972 25554785 PMC4318537

[B61] Zhang Q. Liu L. N. Yong Q. Deng J. C. Cao W. G. (2015d). Intralesional injection of adipose-derived stem cells reduces hypertrophic scarring in a rabbit ear model. Stem Cell. Res. Ther. 6 (1), 145. 10.1186/s13287-015-0133-y 26282394 PMC4539671

[B62] Zhang Z. Kruglikov I. Zhao S. Zi Z. Gliniak C. M. (2021a). Dermal adipocytes contribute to the metabolic regulation of dermal fibroblasts. Exp. Dermatol 30 (1), 102–111. 10.1111/exd.14181 32866299

[B63] Zhang Z. Shao M. Hepler C. Zi Z. Zhao S. (2019a). Dermal adipose tissue has high plasticity and undergoes reversible dedifferentiation in mice. J. Clin. Investig. 129 (12), 5327–5342. 10.1172/JCI130239 31503545 PMC6877341

[B64] Zhao H. Shang Q. Pan Z. Bai Y. Li Z. Zhang H. (2018). Exosomes from adipose-derived stem cells attenuate adipose inflammation and obesity through polarizing M2 macrophages and beiging in white adipose tissue. Diabetes 67 (2), 235–247. 10.2337/db17-0356 29133512

